# Robust Smartphone Assisted Biosensing Based on Asymmetric Nanofluidic Grating Interferometry

**DOI:** 10.3390/s19092065

**Published:** 2019-05-03

**Authors:** Foelke Purr, Max-Frederik Eckardt, Jonas Kieserling, Paul-Luis Gronwald, Thomas P. Burg, Andreas Dietzel

**Affiliations:** 1 Technische Universität Braunschweig, Institute of Microtechnology, 38124 Braunschweig, Germany; max.frederik.eckardt@gmail.com (M.-F.E.); j.kieserling@tu-braunschweig.de (J.K.); paul-luis.gronwald@tu-braunschweig.de (P.-L.G.); a.dietzel@tu-braunschweig.de (A.D.); 2 Max-Planck Institute for Biophysical Chemistry, 37077 Göttingen, Germany; tburg@mpibpc.mpg.de; 3 Technische Universität Darmstadt, Department of Electrical Engineering and Information Technology, 64283 Darmstadt, Germany; tburg@micronano.tu-darmstadt.de

**Keywords:** biosensing, interferometry, portable point-of-care (POC), common mode rejection, nanofluidic, optofluidic grating, smartphone-based

## Abstract

Point-of-care systems enable fast therapy decisions on site without the need of any healthcare infrastructure. In addition to the sensitive detection, stable measurement by inexperienced persons outside of laboratory facilities is indispensable. A particular challenge in field applications is to reduce interference from environmental factors, such as temperature, to acceptable levels without sacrificing simplicity. Here, we present a smartphone-based point-of-care sensor. The method uses an optofluidic grating composed of alternating detection and reference channels arranged as a reflective phase grating. Biomolecules adsorbing to the detection channel alter the optical path length, while the parallel reference channels enable a direct common mode rejection within a single measurement. The optical setup is integrated in a compact design of a mobile readout device and the usability is ensured by a smartphone application. Our results show that different ambient temperatures do not have any influence on the signal. In a proof-of concept experiment we measured the accumulation of specific molecules in functionalized detection channels in real-time and without the need of any labeling. Therefore, the channel walls have been modified with biotin as capture molecules and the specific binding of streptavidin was detected. A mobile, reliable and robust point-of-care device has been realized by combining an inherently differential measurement concept with a smartphone-based, mobile readout device.

## 1. Introduction

In many areas of the world, diseases that are easily treatable from a medical point of view remain a major problem due to a lack of medical infrastructure [1]. Point-of-care (POC) diagnostics enable the rapid and patient-orientated diagnosis of diseases and thus an immediate therapy decision. The aim of POC diagnostics is that it can be used outside of laboratory facilities and is easy to operate even by the patient himself. Consequently, POC need to be portable and robust against changing operating conditions outside of air-conditioned and clean laboratory facilities [2].

Beside a sensitive detection principle, a reliable signal acquisition and evaluation without bulky equipment like computers is crucial. Smartphones are spread worldwide even in developing countries. In 2021 estimated over one third of the world population will use a smartphone (www.statista.com, source: Newzoo) [3]. Additionally, each smartphone is equipped with numerous sensors. Among these, the smartphone camera is one of the most enabling technologies [4]. The resolution of smartphone cameras has improved in the last years to >20 Mpixels [5]. High pixel density and high computing power of modern smartphones allow image recording and processing in real-time. Obtained results can be directly transferred to the next medical center by integrated communication tools like WiFi or Bluetooth. With this, the access to medical services in developing countries or remote areas can be significantly improved.

In combination with microfluidics, new, very sensitive detection technologies have been realized for POC diagnostics [6,7]. Miniaturization enables cheaper disposables, a decrease of the needed sample volume, and shorter process times. In particular, optical biosensors have proven to be powerful tools for the detection of specific molecules in diagnostics, as they are very sensitive and can work in a label-free manner [8,9]. Among those, surface plasmon resonance (SPR) detection is perhaps the most prominent, especially with regards to label-free biomolecule detection [10]. Refractive index changes can be detected with a resolution of $10^{-7}$–$10^{-8}$ RIU (refractive index units) [11,12]. The resonance conditions are very sensitive to changes at the interface due to binding of molecules. Nevertheless, refractive index changes due to protein-protein interaction are relatively small and can be easily overwhelmed by interfering background signals, such as changes in the refractive index of the bulk solution. In particular, temperature fluctuation and nonspecific binding bias the signal, making reference structures essential. Interferometric detection methods do benefit from the combination of detecting and reference structures in the same optical path. Biosensing was successfully realized in a Young interferometer by functionalized waveguides in a microfluidic chip with specific antibodies [13]. One additional wave guide was non-functionalized and serves as reference on the same beam path, accounting for common-mode interference.

Previously, we demonstrated the detection of biomolecules in an optofluidic diffraction grating with a direct background subtraction in a single measurement [14]. The optofluidic chip contains nanofluidic reference and detection channels, which are placed in an interdigitating structure to form an optical grating. The grating is illuminated by a collimated laser and the reflected diffraction pattern is analyzed. Refractive index differences between the two sets of channels can be sensitively detected via an intensity ratio in the diffraction pattern. Importantly, the unit cell of the optical grating has an asymmetric structure which allows direct common mode rejection. Biasing signals due to non-specific binding or temperature fluctuations are suppressed. This is of special interest when working outside of laboratory environments. Our results show that refractive index changes can be measured with a sensitivity of $10^{-5}$ RIU and that the accumulation of biomolecules on the channel wall can be measured in real-time [14]. This makes the asymmetric nanofluidic detector a very promising technology for the implementation in POC.

For the transfer of optical biosensing technologies from the laboratory scale to a mobile POC device, smartphones including digital cameras are a versatile tool to realize cheap and compact mobile platforms. Several reviews reported that SPR-sensors [15,16,17], colometric sensors [18], flow cytometers [19] and other analytical instruments have been realized as POC devices with smartphone readout [4,5,20,21,22,23,24,25,26]. Even though the optical setup was simplified and the used components are cheaper, Lui et al. achieved a sensitivity of $7.4\times 10^{-5}$ RIU and could detect the accumulation of IgG sensitively in a mobile SPR device with a detection limit of 47.4 nM [17].

Also, complex intensity profiles generated by interferometric sensors have been successfully recorded and evaluated by smartphones [27,28,29,30,31]. Hussain et al. [27] evaluated the interference fringes of a microscope glass slide due to angular variations with a smartphone. All components are integrated in a mobile 3D-printed readout device and the image analysis is performed with a homemade application.

Here, we present a portable version of the asymmetric nanofluidic grating-based label-free biosensor that we described previously [14]. The optical measurement setup was transferred to a portable system, where the acquisition as well as the evaluation of diffraction images is realized with the help of a smartphone. We show that a change in refractive index can be measured sensitively by the mobile platform. The asymmetry of the optical gratings allows the reliable measurement even under changing environmental conditions. Surface functionalization of the nanochannels in the optical grating is used to detect the accumulation of specific molecules in a label-free manner.

## 2. Concept

**Figure 1:**
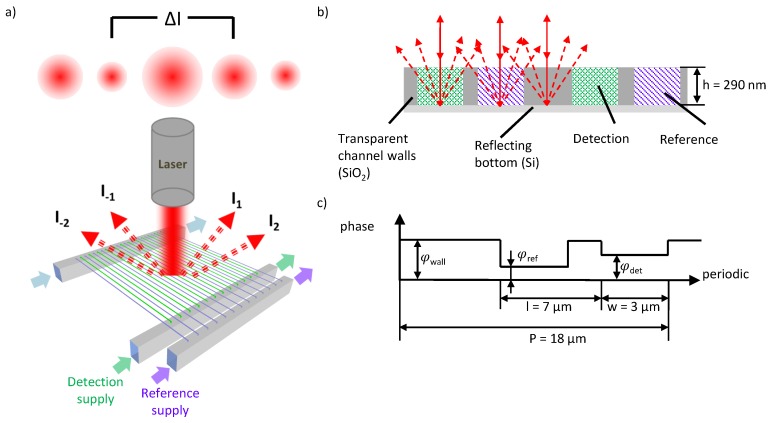
(**a**) Schematic of the asymmetric nanofluidic diffraction grating. Collimated laser light is diffracted. The resulting intensity pattern is asymmetric. Interdigitating structure of reference and detection channels with an asymmetric unit cell of the optical grating. (**b**) Schematic of cross-section of four detection and reference channels. (**c**) Schematic of one asymmetric unit cell in the optical grating and the phase shift related to the area of the grating. P denotes the period of the optical grating, l denotes the asymmetry and w denotes the nanochannel width.

The principle of the asymmetric nanofluidic grating detector has been presented earlier [14]. Briefly, Figure 1a sketches reference and detection nanochannels forming an optical grating. The two sets of nanochannels are arranged in an interdigitating, asymmetric structure. Collimated laser light is diffracted by the optical grating and the resulting diffraction pattern is reflected backwards.

The intensity distribution of the diffraction pattern depends on the phase shift φ between light that passes the nanochannels and light that passes the transparent channel walls (Figure 1c):

(1) φ=2hkn,

Figure 1c describes the asymmetry of the grating and the relating phase shift in the detection channels ($\varphi_{det}$), the reference channels ($\varphi_{ref}$) and the transparent channel walls ($\varphi_{wall}$). Here, P denotes the period of the optical grating, l describes the shift from the symmetric position and w denotes the nanochannel width. As the depth of the channels h is constant as well as the wavelength of the incoming and outgoing light λ (with *k* = 2 π/λ), only the refractive index n of the channels will affect the phase shift and therefore the intensity distribution of the diffraction pattern. The phase shift of the transparent walls stays constant. The asymmetric structure of the grating unit cell gives rise to an asymmetric intensity distribution in case of a refractive index difference between reference and detection channels, $\Delta n=n_{Ref}-n_{Det}\neq 0$. For a symmetric grating the intensity of both the same order maxima would be always equal. The intensity values $I_{m}$ of the single maximum are used to generate the signal $S_{m}$:

(2) $$S_{m}=\frac{I_{m}-I_{-m}}{I_{m}+I_{-m}}=\frac{\Delta I_{m}}{\sum I_{m}}.$$

Equation (2) defines our signal for a pair of maxima at *m* ≠ 0 and equals the standardized difference between two maxima. Previous experiments and analytical investigations have shown that small changes in refractive index between reference and detection channels can be measured with a sensitivity of $10^{-5}$ RIU. However, common changes in the refractive index are inherently suppressed.

## 3. Design and Implementation

### 3.1. Fabrication

A silicon/glass-based fabrication process using silicon-on-insulator (SOI) wafers has been described previously [14]. Here, we reduced the total material costs of chips by about 80% by changing the starting material from SOI wafers to standard silicon wafers. First, an oxide layer with a thickness of h = 290 nm is grown on the wafer surface by a thermal oxidation. The oxide is structed in a lithography process by wet chemical etching with buffered hydrofluoric acid (BHF) to create the nanochannels of the grating. For etching, the buried silicon acts as an etch stop, creating nanochannels with a depth equal to the oxide thickness. Besides low required sample volume, the channel depth in the nanometer range ensures that changes in the refractive index on the channel surface due to protein accumulation, contribute considerably to the optical path length through the channel. Additionally, protein binding to the channel walls is not diffusion limited and therefore a complete depletion in the sample volume can be achieved.

**Figure 2:**
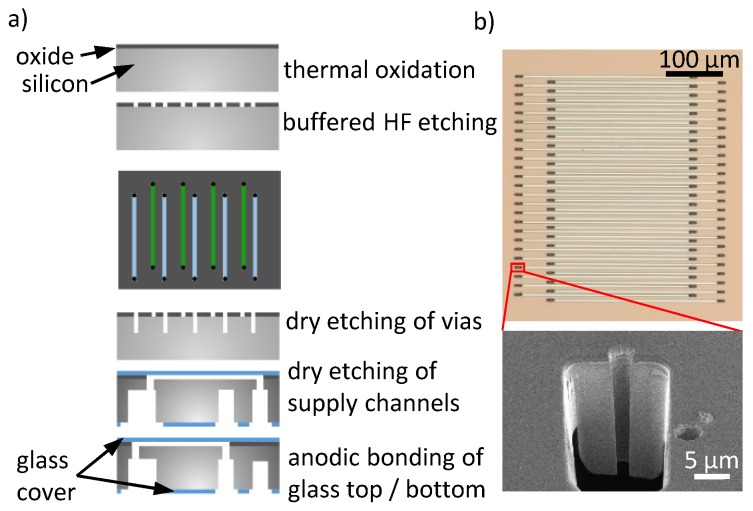
(**a**) Schematic illustrating the fabrication. (**b**) Optical micrographs of the nanofluidic grating. At the end of each nano channel, a via connects the channel to bigger supply channels. Also shown is an electron microscopy micrograph of a focused ion beam (FIB) cut through one via.

Each nanochannel was connected to bigger supply channels by small vias. The small vias allow us to address only the detection channels without affecting the reference channels. We opened 3 μm vias by deep reactive ion etching (DRIE) and connected the nanochannels with bigger supply channels from the bottom side, which have also been processed by DRIE. Finally, all fluidic channels were sealed with Boroflaot 33 wafers by anodic bonding. The top glass wafer, sealing the optical grating, had a thickness of 200 μm. The bottom glass wafer had a thickness of 700 μm. Before bonding, through glass vias were opened by a femtosecond laser ablation to connect the chip to the fluidic interface. Figure 2 shows a microscope image of the optical grating consisting of nanochannels. Each channel has a width w = 3 μm and a length of 320 μm. In this study, we used a chip with an asymmetry of l = 7 μm and a grating period of P = 18 μm (cf. Figure 1).

### 3.2. Mobile Optical Setup

Many individual solutions have been found to integrate smartphones, microfluidics and additional measurement equipment in a compact and robust way. 3D-printing/fused-filament fabrication enables researchers to give their POC device an appropriate frame while still in research [27,32].

**Figure 3:**
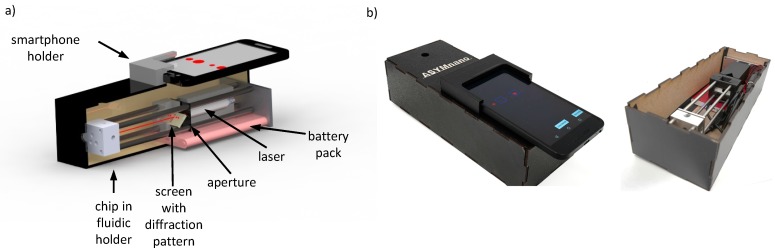
(**a**) CAD (computer-aided design) drawing of mobile setup. (**b**) Picture of mobile readout device closed with smartphone (left) and opened showing the optical path (right).

The basic setup of our portable optical readout device is sketched in Figure 3. A collimated laser diode module with a power of 1.2 mW, a wavelength of λ = 635 nm and a beam diameter of 2.9 mm (CPS635R, Thorlabs, Newton, NJ, USA) passes through an aperture (diameter = 1 mm) and a 3 mm slit in the screen center until it enters the chip at the opposite side of the device. The laser light was diffracted and reflected backwards. The resulting diffraction pattern was caught on a polystyrene screen, which was placed at an angle of 45${}^{\circ }$ to the beam. The camera of the smartphone was placed directly above the screen to take images of the diffraction pattern. The laser was powered by a 5 VDC USB battery pack (CSP2, Thorlabs, Newton, NJ, USA) to be independent from the electrical supply. The laser was fixed in a 3D-printed housing made of acrylonitrile butadiene styrene (APS) (Ultimaker 2+, Geldermalsen, The Netherlands). The housing and all other components were placed in an optical cage system with four rods ensuring the position of all components on the optical axis. Also the housing of the screen and the holder for the smartphone were 3D-printed parts made of APS. The cage system and the fluid connector for the chip have been fabricated out of aluminum. The chip was covered by black masking tape, leaving only the part with the grating open, so that light from the surrounding, biasing the signal, was suppressed. In this way we were able to use a laser source without the need of focusing or additional alignment of the grating and the laser. This simplifies the setup and the handling significantly. Furthermore, the slit in the center of the screen allowed us to get rid of the zero-order maximum, which is very intense compared to the other maxima but contains no relevant information. The entire optical system was placed in a black box to block light from the surroundings. Only small areas for the fluidic connection and for the smartphone camera were left open. The position of the smartphone on top of the screen was fixed by a 3D-printed mount. Figure 3 shows a CAD (computer-aided design) drawing of the optical setup and a picture of the portable test cassette, respectively.

### 3.3. Smartphone Application

**Figure 4:**
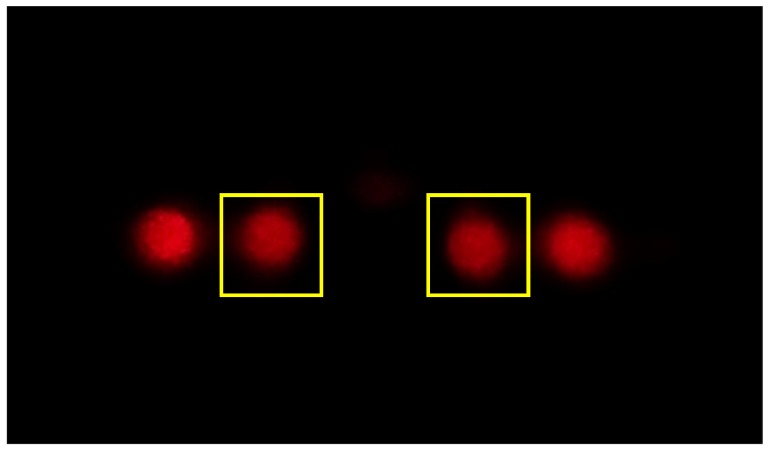
Diffraction pattern recorded via the smartphone application. After calibration and recording, the region of interest is placed around the maxima to be evaluated.

The image acquisition and evaluation takes place via smartphone. For this purpose, an Android application was created that controls the camera of the smartphone and also processes the images. For the user it is difficult to find the appropriate settings of the camera. Also white balance or other automatic features of the smartphone software might bias the result. Some POC devices using the smartphone cameras try to overcome this limitation with reference areas on the image. Our Android application guides the user through the test procedure and a self-running calibration is performed before the start of the experiment. During the calibration, several images are taken automatically with different camera settings (ISO, exposure time). The settings that are most suitable for the lighting conditions and the camera sensor are selected in order to enable an optimal test exposure. On the one hand the pixel values must not exceed the saturation value of the smartphone camera (here 8 bit) and on the other hand the whole dynamic range of the camera sensor should be used, Therefore, the exposure time was set to a value close to saturation with a margin of minimum 20 grey values. The ISO value was set as low as possible to minimize noise. Figure 4 shows a diffraction pattern taken with the smartphone camera after calibration. It shows four maxima of the diffraction pattern. The zero-order maximum is filtered out due to the slit at the screen center.

First, a series of images was taken in the reference state when reference fluid fills the entire grating. The average signal was set to zero to generate a baseline. The user was then prompted to enter the sample and to continue the measurement afterwards.

In addition to ISO settings and exposure time, the time between two images and the number of images can be defined. In the experiments described here, an interframing time of 2.5 s and 20 images for each measurement section were selected. The images were then saved and the user can define positions of the maxima to be evaluated by a simple double-click on each maximum. Within a certain region of interest (250×250 pixel) around each maximum all pixel values were integrated. Finally, the signal was calculated according to Equation (2). The reference state then yields the baseline and the average difference from the second measurement part to the baseline gives the relevant signal strength. For comparison, the recorded images were also analyzed with a Matlab program. The moving average of the signal was calculated (window length = 10).

To use the smartphone application, a late version of an Android smartphone (Nougat 6.0/API Level 23) and a camera with a relatively large number of megapixels is required. The hardware must also support Google’s camera2api.

## 4. Materials and Methods

### Fluidics

**Figure 5:**
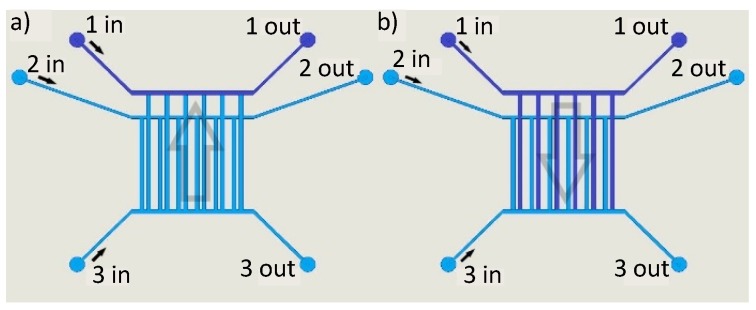
Fluidic control in the optofluidic grating. All fluids enter the chip via supply channels. Supply channels 1 and 2 address reference and detection channels separately. Supply channel 3 is connected to both sets of nanochannels. By controlling the pressure levels in the supply channels, a constant flow through the supply channels is achieved and the grating is filled with reference (**a**) or sample fluid (**b**).

As described above, the chip design allows us to run different fluids through the detection and reference channels at the same time as the two sets of channels are connected to separate supply channels. Figure 5 shows the fluidic setup of the chip. Through channels 1 and 2 reference and detection channels can be filled independently. Through channel 3 both sets of nanochannels can be flushed with the same fluid for wash out.

To control the fluidic flow, pressure controllers were used. In real life application, pressure controllers are not appropriate, as they need supply to gas pressure and additional computing power. However, for characterizing the whole setup this method was beneficial. By adapting the pressure level in channels 1–3, the fluidic flow inside the grating can be controlled very precisely.

A schematic of the fluidic flow is given in Figure 5 and Table 1 gives an overview on the applied pressure levels for washing/reference state and measuring/detection state.

For the trials presented here, we used an aluminum chip connector with fittings for the fluidic access as shown in Figure 3.

#### Test Solutions

For characterization of our readout device with a nanofluidic chip, we first measured the signal dependency on refractive index differences between reference and detection channels by pushing glycerol solutions with a concentration range of 1–14% (*w*/*w*) in deionized (DI) water, equivalent to refractive index range of n = 1.334–1.35, through the detection channels. The reference was filled with DI water during the entire measurement. Before each measurement, the refractive index of the glycerol solutions were measured with a handheld refractometer (DR201-95, A.Krüss, Hamburg, Germany) for comparison. All fluids were filtered before entering the chip to avoid clogging. Initially, the grating was filled with DI water and the resulting signal taken as a baseline. In the second step, glycerol solution was pushed through the detection channels for one minute before the signal recording started again. Each measurement was performed multiple times.

Also for the functionalization of the detection channels with PLL-PEG-biotin (poly-l-lysine(20)-g[3.5]-polyethylene glycol(2)/polyethylene glycol(3.4)-biotin(50%), SuSoS, Dübendorf, Switzerland) the pressure controllers were used. PLL-PEG-biotin (0.125 mg/mL) in phosphate buffered saline (PBS) was pushed through the detection channels for 30 min (via channel 1). Here, the fluid in the reference channels was pure PBS. In the second step, streptavidin (0.05 mg/mL in PBS, Thermo Fisher Scientific, Waltham, MA, USA) was pushed through all channels.

## 5. Results and Discussion

### 5.1. Characterization

**Figure 6:**
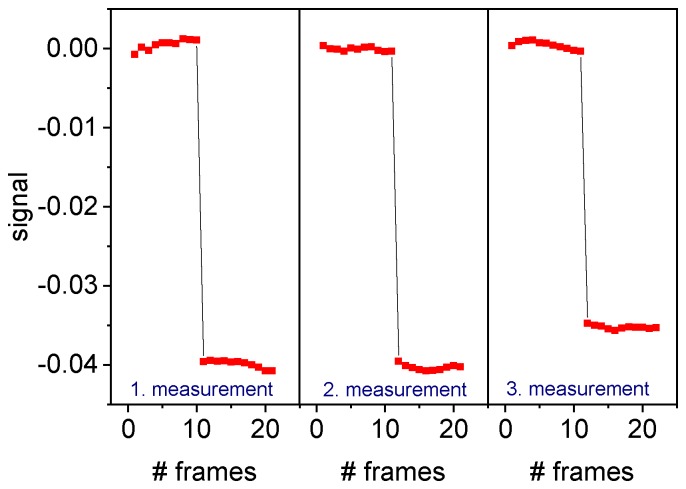
Signal for three consecutive measurements with a 14% glycerol solution in DI water. The reference fluid is DI water.

We first performed calibration measurements with different glycerol solutions in DI water. In the first part of the measurements all channels were filled with DI water to determine the baseline signal. Afterwards, the glycerol solution was pushed through the detection channels for one minute before the second part of the measurement started. The signal was evaluated based on the first order maxima. The same procedure was then repeated at least three times for every concentration of glycerol. Figure 6 shows one exemplary set of measurements for a glycerol concentration of 14% (w/w), which is equivalent to a change in refractive index Δn = 0.0188 RIU. Even though some variation within the measurement and from measurement to measurement can be observed, the results demonstrate that the signal acquisition was repeatable and that the system was stable within a certain variance.

**Figure 7:**
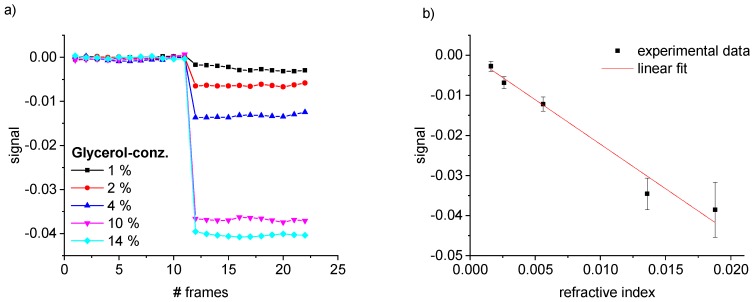
Signal of the first mode maxima and its dependency on refractive index changes. (**a**) Plot of the signal vs. number of consecutively recorded frames. For the first 11 frames, DI water ran through all channels and the resulting signal was set as a baseline. In the following phase, DI water with different glycerol concentrations was injected into the detection channels. (**b**) Calibration curve as a function of Δn.

Figure 7a shows how the measured signal is affected by a change in refractive index.

In Figure 7b, the signal values are plotted over the independently measured Δn. The signal decreased with the refractive index difference. The dependency can be described with a linear regression with high evidence (correlation coefficient R = 0.986). The slope of the linear regression corresponds to the sensitivity of our mobile device. With a sensitivity of $s=\partial S_{2}/\partial \Delta$n = −2.22 RIU${}^{-1}$, the measured sensor response is below the former reported values [14]. The refractive index equivalent of the standard deviation for the first 11 frames is also one order of magnitude higher ($\sigma_{\Delta n}=1.12\times 10^{-4}$ RIU) compared to results obtained with the non-mobile device [14]. Most probably intensity losses due to the monitoring on a screen instead of measuring the light intensities directly on a camera sensor might be a reason. Additionally, the mobile setup was more susceptible to vibrations compared to an installation on an optical table and contains less stable optical components (e.g., no temperature controlled laser diode, non-cooled camera) which contributes to the higher losses. However, the calibration measurements show that a change in refractive index can be measured sensitively and reliable in the mobile setup.

### 5.2. Common Mode Rejection

**Figure 8:**
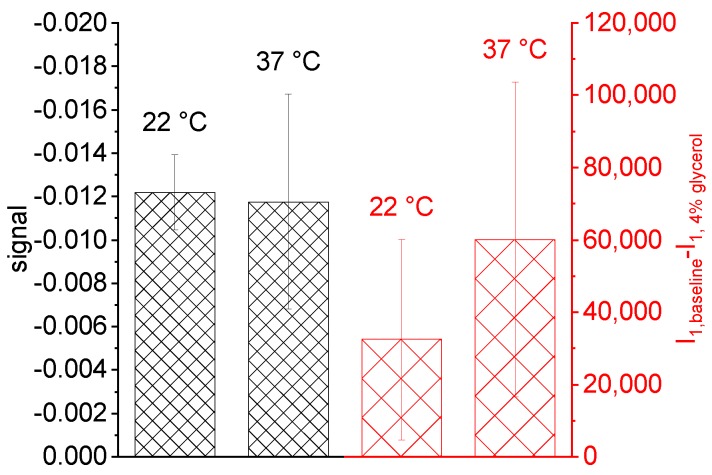
Signal and intensity difference for two different temperatures. The experiments were run with 4% glycerol solution and DI water as reference in an incubator. First at room temperature, second at 37 ${}^{\circ }$C.

One aim of POC devices is to enable medical support independent of laboratory facilities or medical environment. As the refractive index is very sensitive to changes in temperature, adequate common mode rejection between the detection and reference channels is essential to avoid misinterpretation of the signal. Here, we show that the asymmetric structure of our nanofluidic grating allows the direct optical subtraction of such common changes without the need for additional reference measurements. We performed several measurements with 4% glycerol solution and DI water as reference at room temperature (22 ${}^{\circ }$C). Afterwards we placed the complete setup into an incubator and waited until the setup had reached a temperature of 37 ${}^{\circ }$C. We then repeated the glycerol/water measurements under the higher temperature conditions and plotted the signal as well as the difference in intensity of a single maximum between baseline and the high refractive index phase for both temperature levels, $I_{1}$(baseline) −$I_{1}$(4% glycerol), in Figure 8. The intensity of the single maxima shows a clear shift due to the change in temperature indicating the effect of temperature on the refractive index. However, the left part of Figure 8 shows that the impact of temperature can be suppressed in the measured signal. The signal shows very similar values for both temperature levels, even though the standard deviation increases for the higher temperature. This might again be due to the fact that the optical path was not temperature-controlled. Nevertheless, the average difference between the signal of both temperature levels $S_{1}\left(T_{1}\right)-S_{1}\left(T_{2}\right)$ corresponds to 0.0004 RIU, which is one order of magnitude smaller than the change in refractive index of water from temperature T1 to T2 [33].

### 5.3. Protein Detection

**Figure 9:**
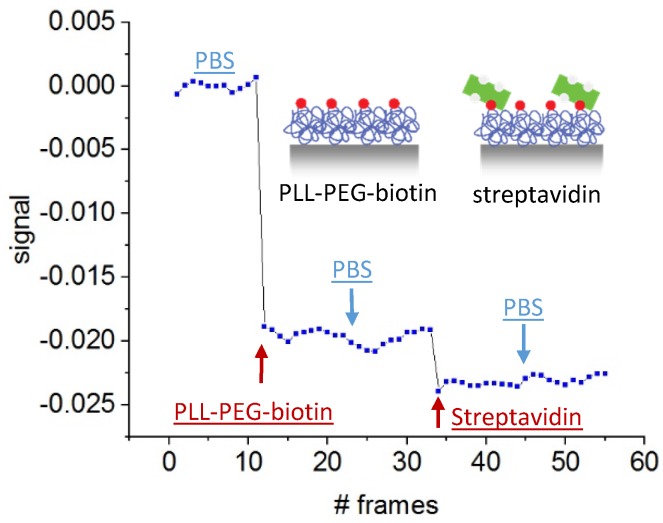
Functionalization and detection of poly-l-lysine(20)-g[3.5]-polyethylene glycol(2)/polyethylene glycol(3.4)-biotin(50%) (PLL-PEG-biotin) in detection channels.

For bulk refractive index sensing, the low channel depth is not beneficial as the light-matter interaction length is very low. This results in a poor sensitivity compared to commercial bulk refractive index sensors. However, for the detection of surface-bound layers inside the channels, the nanosized channel depth is a big advantage. In Figure 9 we show, that we were able to functionalize the detection channels with PLL-PEG-biotin and to detect the specific accumulation of streptavidin afterwards. We split the experiment into four measurements as the smartphone application only records images for the reference phase and the sample phase before the next measurement starts. In the first measurement, we ran PBS through all channels to find the baseline. Afterwards, we pushed the PLL-PEG-solution through the detection channels for 30 min before recording the second set of images with the smartphone application. Due to electrostatic interactions, the PLL adhered to the channel surface. This is also visible in the plot. The signal shows a clear shift due to the accumulation of capture molecules. Even after washing with PBS through channel 3 in the second measurement, the signal remained constant, indicating the formation of a stable protein layer.

**Figure 10:**
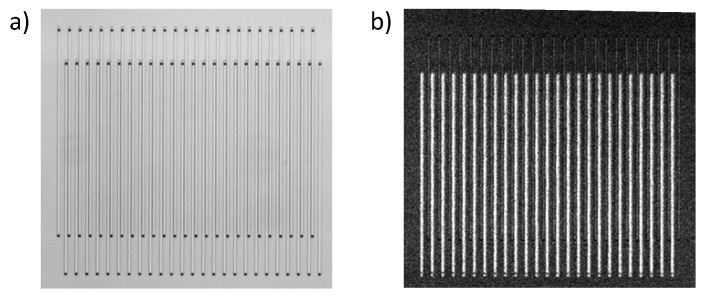
Image of nanofluidic grating after binding of fluorescence labeled streptavidin in functionalized detection channels. (**a**) bright field (**b**) fluorescence image.

Streptavidin was then introduced to the reference and detection channels (channel 1 and channel 2). Streptavidin attached reliably to the biotin-derivatized surfaces. This was validated using the same experimental protocol but with fluorescence labeled streptavidin as a read-out (Figure 10). The fluorescent image showed that streptavidin binds mainly to every second channel, which had been functionalized with PLL-PEG-biotin before.

The corresponding label-free experiment in Figure 9 revealed via a shift in the interferometric signal that streptavidin binds in the detection channels, and that our portable, smartphone based asymmetric nanofluidic device is able to detect it. In the last measurement set, again PBS was pushed through all channels, but the signal stayed constant due to the unaffected protein layer.

Figure 9 represents the signal shift due to streptavidin binding in pure PBS solution. However, in real point-of-care application the sample will be contaminated with other components as they appear in urine or blood. Even after sample filtration to avoid nanochannel clogging, unspecific binding cannot be eliminated, indicating the importance of reference structures. In the asymmetric grating consisting of detection and reference channels, disturbances of the signals by non-specific binding are suppressed in the measurement. Additionally, the channel depth in the nanometer range allows the complete depletion of target molecules in the sample. Therefore, the change in signal due to protein binding depends mainly on the functionalization density on the detection channel surfaces and on the sample volume pushed through the grating. We expect that significantly lower concentrations of protein can be measured provided that the accumulation time is extended or the flow rate is increased.

### 5.4. Concept for POC Loading

**Figure 11:**
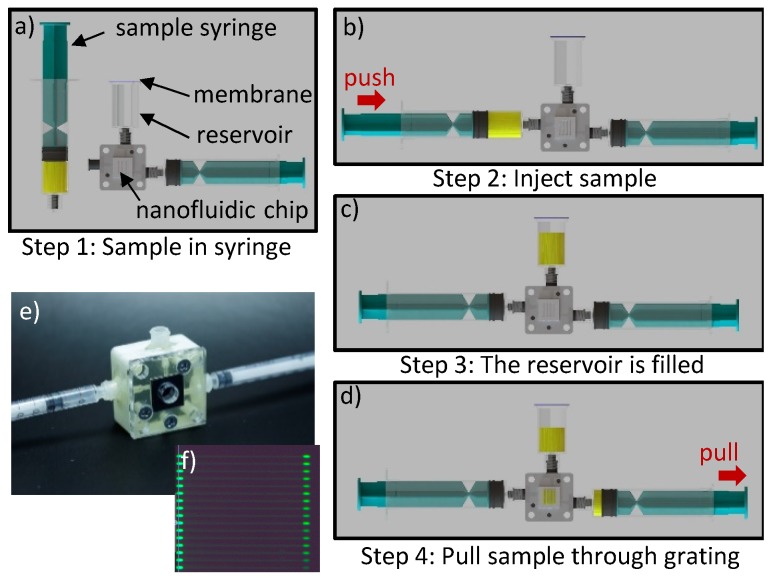
(**a–d**) Concept of the simplified fluidic connector. Introduction on how to apply the sample. (**e**) Image of the simplified fluidic connector made by 3D printing. (**f**) Optofluidic grating filled with fluorescence dye.

In a true POC setting, the chip holder as well as the chip itself will be contaminated with sample material of the patient and needs to be disposable after each measurement. Hence, a complicated pressure control system with 6 in- and outlets is not user-friendly and also not necessary. Under the assumption that the chips will be functionalized with specific antibodies in the fabrication process before, the user just needs to push the sample into the chip while exchange of volume inside the grating has to be ensured. In case compatible antigens are present in the sample, these will be captured by the functionalized antibodies in the detection channels and therefore alter the refractive index difference between reference and detection channels. We designed a new simplified fluidic holder for this application scenario, which is shown in Figure 11. The connector has just two in- and outlets and one reservoir. The reservoir is sealed with a hydrophobic membrane, which allows the exchange of air but prevents the leakage of contaminated sample fluid. The in-and outlets have luer connectors enabling the injection of samples by commercial syringes. In the next step, the user needs to generate a vacuum by simply pulling an empty syringe. This will pull the sample through the grating and ensures an adequate volume exchange. The connector prototype is made by 3D-printing in an AGILISTA-3200W (Keyence, Osaka, Japan). Figure 11 shows a simplified chip holder and sketches how the user can easily introduce the sample fluid. The functionality of the holder has been proven with fluorescence dye, which has been introduced with a syringe into one supply channel and subsequently pulled through the grating with a second syringe (Figure 11f).

## 6. Conclusions

We presented the transfer of an optical biosensing platform from the lab scale to a mobile POC setup. The detection principle is based on measuring a change in refractive index between two sets of nanochannels forming an optical grating.

Smartphones are widespread, versatile tools featuring substantial computing power and a collection of sensors. Here, the smartphone has been used to record and analyze the diffraction pattern in a user-friendly environment. The smartphone-based interferometric platform has been successfully applied to determine refractive index changes with a detection limit of 1.12 × 10${}^{-4}$ RIU. Also the accumulation of specific molecules in the detection channels was sensitively detected by functionalizing the detection channel walls with biotin and detecting the specific binding of streptavidin accordingly. However, the application of low-cost components, like laser and smartphone camera, or the low stability of the portable device compared to a measurement on an optical table, also bears some drawbacks we need to account for. (1) The signal stability is inferior to the non-mobile setup by one order of magnitude, but is still high enough to detect protein binding. (2) Most of the world population have access to smartphones and know how to use them. Nevertheless, the variance between smartphones is high. Different camera qualities and designs make the implementation and handling with the smartphone application difficult. Fingerprints or scratches on the camera lens might bias the signal additionally [21]. Consequently, we believe that a smartphone is a powerful tool to realize cheap, easy-to-use and available POC setups. However, due to the impact of different smartphones from different users, we propose to have one smartphone with the setup instead of using the smartphone of the individual user/patient to guarantee the quality of the POC device. Other biasing signals, like non-specific binding or temperature fluctuations are suppressed in the measurement itself due to the asymmetric structure of the optical grating. Hence, our new smartphone based POC platform enables a robust and sensitive detection of specific molecules in an interferometric measurement.

## Figures and Tables

**Table 1:** Applied pressure levels to guide fluidic flow through the grating.

Step	Channels 1 and 2, in/out	Channel 3, in/out
wash out	0.2/0.1 bar	0.5/0.3 bar
sample	0.5/0.3 bar	0.2/0.1 bar

## Abbreviations

The following abbreviations are used in this manuscript:

ABS	acrylonitrile butadiene styrene
CAD	computer-aided design
DI	deionized water
h	nanochannel depth
I	integrated intensity of a maximum
k	wave number
l	asymmetry
ISO	international organization for standardization
λ	wavelength
n	refractive index
m	maximum mode
P	period of the optical grating
PBS	phosphate buffered saline
φ	optical phaseshift
PEG	polyethylenglycol
PLL	poly-l-lysine
RIU	refractive index unit
S	signal
SPR	surface plasmon resonance
T	temperature
w	nanochannel width
